# Continuous Theta Burst Stimulation over the Left Dorsolateral Prefrontal Cortex Decreases Medium Load Working Memory Performance in Healthy Humans

**DOI:** 10.1371/journal.pone.0120640

**Published:** 2015-03-17

**Authors:** Nathalie Schicktanz, Matthias Fastenrath, Annette Milnik, Klara Spalek, Bianca Auschra, Thomas Nyffeler, Andreas Papassotiropoulos, Dominique J.-F. de Quervain, Kyrill Schwegler

**Affiliations:** 1 University of Basel, Department of Psychology, Division of Cognitive Neuroscience, Basel, Switzerland; 2 University of Basel, Department of Psychology, Division of Molecular Neuroscience, Basel, Switzerland; 3 University of Basel, Department Biozentrum, Life Sciences Training Facility, Basel, Switzerland; 4 University of Basel, Psychiatric University Clinics, Basel, Switzerland; 5 Departments of Neurology and Clinical Research, Perception and Eye Movement Laboratory, Inselspital, Bern University Hospital, and University of Bern, Bern, Switzerland; 6 Center of Neurology and Neurorehabilitation, Luzerner Kantonsspital, Luzern, Switzerland; University of California, Merced, UNITED STATES

## Abstract

The dorsolateral prefrontal cortex (DLPFC) plays a key role in working memory. Evidence indicates that transcranial magnetic stimulation (TMS) over the DLPFC can interfere with working memory performance. Here we investigated for how long continuous theta-burst stimulation (cTBS) over the DLPFC decreases working memory performance and whether the effect of cTBS on performance depends on working memory load. Forty healthy young subjects received either cTBS over the left DLPFC or sham stimulation before performing a 2-, and 3-back working memory letter task. An additional 0-back condition served as a non-memory-related control, measuring general attention. cTBS over the left DLPFC significantly impaired 2-back working memory performance for about 15 min, whereas 3-back and 0-back performances were not significantly affected. Our results indicate that the effect of left DLPFC cTBS on working memory performance lasts for roughly 15 min and depends on working memory load.

## Introduction

Working memory (WM) is a process allowing temporary storage and manipulation of information [1]. Studies in animals have shown that WM processes depend on the excitability and sustained firing of neurons in the dorsolateral prefrontal cortex (DLPFC) and other cortical regions [2]. WM processes in humans can be investigated by neuroimaging techniques such as functional magnetic resonance imaging (fMRI) [3, 4]. However, fMRI results are of correlational nature. A method that examines the task-critical role of neural activity is transcranial magnetic stimulation (TMS) that interferes with task-relevant neural activity [5, 6]. TMS can be applied using single pulses or a train of pulses. The latter is referred to as repetitive TMS (rTMS) that can induce TMS aftereffects that outlast the stimulation period [5]. Application of rTMS can either be offline, when rTMS is applied prior to a task or online, when both stimulation and cognitive performance occur simultaneously. While online studies can provide information regarding to when a brain region is active within a process, the advantage of an offline approach is that nonspecific disruption of performance due to concurrent discomfort, stimulation noise and muscle twitches is avoided [5]. However, it is crucial for offline TMS studies or combined offline TMS-fMRI studies to have an estimation of the duration of the aftereffect for a specific phenotype, as this period should ideally last throughout the subsequent task [5].

The facilitating or inhibiting aftereffects of offline rTMS on neuronal excitability were mainly assessed for the motor cortex. Low frequencies rTMS (≤ 1 Hz) decrease motor cortical excitability, as assessed by motor evoked potentials in hand muscles (MEP), whereas high frequencies rTMS (> 1 Hz) increase motor cortical excitability [7]. In standard rTMS, pulses are applied steadily and with an identical stimulus interval [5]. In contrast, the theta burst stimulation (TBS) by Huang et al. [8] consists of a burst of 3 pulses, which are applied at 50 Hz. The triplet of pulses is repeated every 200 ms. TBS was shown to decrease or increase motor cortical excitability assessed by MEPs depending on the temporal application of these bursts. In the case of continuous TBS (cTBS) that decrease excitability, a 40 s train of uninterrupted TBS is given with a total of 600 pulses. In contrast, intermittent TBS (iTBS) refers to the application of a 2 s train of TBS that is repeated every 10 s for a total of 190 s and 600 pulses and was shown to increase motor cortical excitability. There is some evidence for prolonged inhibitory aftereffect durations with a cTBS paradigm, as compared to 1 Hz rTMS. The aftereffect duration of standard low frequency rTMS is lasting approximately half the duration of the stimulation train [9]. In direct comparison with 600 pulses of 1 Hz rTMS, 600 pulses of cTBS affect eye saccade latencies for up to 30 min, while the effect of 10 min of 1 Hz rTMS lasts for about 8 min [10]. Furthermore, in the original TBS article by Huang et al. [8], motor cortical excitability is suppressed for 60 min after a total of 600 pulses.

Previous studies using an offline rTMS approach have indicated that stimulating the prefrontal cortex (PFC) can interfere with WM accuracy. These offline rTMS studies used 2 types of WM tasks, one of which is the n-back task. Within this task, subjects are requested to monitor a series of stimuli and are required to indicate when the currently presented stimulus is the same as the one presented n trials before [11]. The other WM task is the delayed stimulus recognition task using spatial and facial stimuli. In this context, Mottaghy et al. [12], using a 1 Hz rTMS, found increased error rates in this task in the first 5 min after TMS. No information regarding aftereffect duration is available in the following studies. In a related type of task, Morgan et al. [13] used a cTBS protocol. Participants had to mentally combine either the colors, or orientations, or both colors and orientations of 2 geometrical shapes and had to decide whether or not a delayed single test stimulus was a combination of the 2 shapes presented before. rTMS led to decreased accuracy only in the combined condition. A third TMS study used cTBS and a modified 2-back task with faces, or scenes, or a combination of both and found decreased accuracy performances both in the faces and the combined condition but not in the scenes condition [14]. Of the 4 TMS studies described here, only one used a classical n-back task. That study used the 0- and 2-back letter task and a 1 Hz rTMS approach and found a significant interaction between stimulation condition (sham or real TMS) and WM-load (0-back, 2-back) on accuracy [15] only after controlling for individual resting motor threshold (rMT). In addition to WM, rTMS over the PFC was also used to investigate other types of memory, for example declarative memory [16].

It is not known for how long cognitive behavioral effects of cTBS interventions are lasting. Here we aimed at investigating the aftereffect duration of a 50 Hz cTBS protocol [8] over the left DLPFC in a classical n-back WM letter task as well as the interplay between the TMS effect and distinct levels of WM-load in healthy subjects. We expect a time-dependent decrease in performance after TMS in the 2-back and 3-back condition, but not in the 0-back condition, compared to sham cTBS stimulation. As there is some evidence for prolonged aftereffect durations for cTBS paradigms as compared to 1 Hz rTMS, we expect a longer cTBS aftereffect on performance than previously observed using 1 Hz rTMS [12].

## Methods

### Participants

Forty healthy, right-handed subjects (20 males, mean age 24.30, range 18–39; 20 females, mean age 22.55, range 18–31) participated in our study. Handedness was assessed by the Edinburgh Handedness Inventory [17]. Participants were free of any psychiatric and physiological disease and did not report any case of epilepsy among first-degree relatives. The local ethics committee (Ethikkommission Nordwest- und Zentralschweiz EKNZ) approved this study. All participants gave written informed consent prior to participation and received 25CHF/h as compensation for their participation.

### Transcranial magnetic stimulation target identification

**Fig 1:**
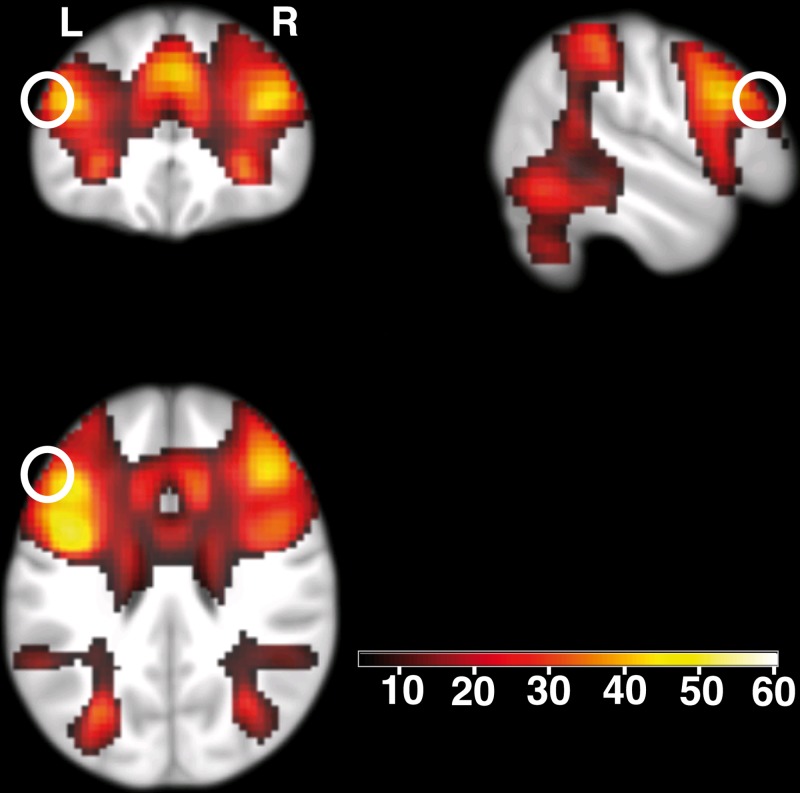
Brain activity related to working memory. Prefrontal activity, color-coded t values (*p*_*whole-brain-FWE-corrected*_ *< 0.05*; N = 707). The white circles depict the area within the left dorsolateral prefrontal cortex stimulated by TMS (centered at [–53, 31, 25] in Montreal Neurological Institute space). Activations are overlaid on coronal (top left), sagittal (top right), and axial (bottom left) sections of a study-specific group template. L, left side of the brain; R, right side of the brain.

The stimulation location over the left DLPFC was derived from an independent sample of over 700 subjects performing a similar WM task during fMRI [18]. The DLPFC target region for TMS was identified based on the results of a 0- and 2-back fMRI study [18]: We identified the peaks of a large activation cluster in the DLPFC (see Fig. 1): Montreal Neurological Institute space (MNI) [-44, 25, 28], T = 50.13, family-wise error corrected for the whole brain *p* < 0.05. Right hemisphere: MNI [41, 33, 28], T = 44.41, family-wise error corrected for the whole brain *p* < 0.05. We chose the left rather than the right DLPFC as a target for TMS as the left DLPFC appeared to be slightly stronger associated with the n-back task. Because the TMS coil (figure-of-eight) can only reach the cortex surface, i.e. areas no deeper than 2–3 cm from the skull surface [5], we selected a proximate coordinate that was close to the peak and close to the cortex surface (MNI [–53, 31, 25], T = 28.13, corresponding coordinate in Talairach space [–50, 25, 28], Brodmann area 9 according to Talairach Client [19]).

In the next step a frameless stereotaxic software (Brainsight, Rogue Research Inc., Montreal Canada) was used to target the WM specific peak voxel activation by means of a navigated TMS coil. Because of the large extent of the identified brain activation clusters, we used an ICBM 152 brain template as provided in the SPM software package (http://www.fil.ion.ucl.ac.uk/spm/software/spm8/).

### Transcranial magnetic stimulation and neuronavigation

TMS was performed with a biphasic magstim Rapid2 stimulator (The MAGSTIM Company Ltd, Whitland, UK). rMT was recorded with a standard 70 mm figure-of-eight coil. As described before [20], we defined rMT as the lowest stimulation intensity required to induce any visible twitch in the right hand in at least 5 out of 10 trials, when applied over the left primary motor cortex. For active cTBS stimulation an air-cooled 70 mm figure-of-eight coil was applied. This specific coil was navigated and positioned in the same way during the sham condition, but then was not connected to the TMS system. In order to create comparable background noises, the independent cooling system was turned on and the connected sham coil simultaneously imitated the characteristic clicking sound.

Active or sham cTBS stimulation was delivered to the left DLPFC (MNI coordinates: [-53, 31, 25) with 80% of individual rMT. The coil was navigated to the target region and positioned tangentially to the skull with the coil-handle pointing backwards at an angle of 45 degrees to the corresponding parasagittal line. Thus, the current direction in the brain for the first half-wave of the biphasic pulse was in a posteriorlateral-to-anteriomedial direction. cTBS parameters were adopted from Huang et al. [8]. In brief, TMS consisted of 50 Hz trains of 3 TMS pulses repeated every 200 ms continuously over a period of 40 s (600 pulses total).

### Procedure

At the beginning state-anxiety was assessed with the Spielberger State Anxiety Inventory (STAI) [21]. Additionally, the question as to how much anxiety the subjects experienced at this moment had to be answered on a continuous visual analog scale (VAS), ranging from 0 (no actual anxiety at all) to 100 (highest anxiety imaginable). Subsequently, participants were instructed and then trained on the n-back task. The n-back main task started if the subject’s responses in the 0- and 2-back training task were correct in at least 75% of all cases, otherwise the training was repeated a second time. After the n-back main task, subjects were asked again to indicate their anxiety on the VAS, followed directly by rMT assessment, then again indicating their anxiety on the VAS, both before and after TMS. After TMS the participants took a seat at a table for the second n-back session, implicating a delay of approximately 3 min between stimulation and task. Subsequently participants completed the STAI, indicated their anxiety on the VAS, and filled in a terminal questionnaire assessing their general commitment to the study. Finally, participants stated if they believed to have been assigned to the active or sham stimulation group. The total length of the experimental procedure was approximately 105 min.

### Behavioral tasks and questionnaires

The 0-, 2-, and 3-back version of the n-back letter task [22] was used. One n-back session consisted of 6 blocks. Each block contained one 0-, one 2-, and one 3-back sequence in random order. Therefore, participants completed 18 sequences—or 6 blocks—both before and after TMS. One block lasted 3 min and therefore one n-back session lasted 18 min. In the 0-back condition participants had to respond to the letter “x”. This served as a non-memory-guided control, measuring general attention and does not require the manipulation of information within WM. In the 2-back condition participants had to compare the currently presented letter with the one presented 2 steps before, and in the 3-back condition with the one presented 3 steps before, and had to indicate whether they were identical or not. The 2-back as well as the 3-back conditions require online monitoring, updating, and manipulation of remembered information and therefore are assumed to involve key processes of WM [11].

The primary variable of interest was accuracy (ACC: hits plus correct rejections divided by the total number of letters shown), a widely used measure of WM performance. To illustrate the change of performance after TMS, we calculated delta values for each block after TMS according to the formula: Delta n-back performance after TMS in block X = (n-back performance in block X after TMS multiplied with 100) / (n-back performance in the last block before TMS). As the mean over all 6 blocks before TMS would not account for inter-individual differences in the learning effect that was present across the 6 blocks, we used the last performance before TMS in our model.

A value higher than 100 indicates an improved performance while a value lower than 100 indicates an impaired performance in the actual block compared to the performance in the last block before TMS. D-prime analysis was used as an additional variable of interest and represents the Z-transformed values of hit rates minus false alarm rates. D-prime takes the subjects response bias into account, that is the general tendency to respond target or nontarget [23].

### Statistical analysis

Analyses were done in R (version 3.0.2; R Development Core Team 2012). Delta ACC n-back performance differences between stimulation groups (sham cTBS or active cTBS) were analyzed using linear mixed models (nlme-package) in combination with ANOVA (SS II). Subject was included as the random effect of the mixed model. The independent variables of interest were the stimulation group, the WM-load and the interaction between stimulation group and WM-load. Sex, age, and the interaction of WM-load with sex and age were included as covariates of no interest. A nominal alpha level of 0.05 was chosen for all statistical tests. In case of a significant interaction effect of stimulation by WM-load (0-, 2-, 3-back), a *post-hoc* analysis was applied separately for each WM-load to analyze the source of the significant interaction. For the *post-hoc* tests, we used a Bonferroni correction for multiple testing controlling for 3 independent tests (0-, 2-, 3-back).

A Fisher exact test was conducted to examine the association between the stimulation group and the subject's belief on which stimulation they received. Group differences in anxiety reported on the VAS, STAI, rMT, age, handedness, time of day, and motivation were analyzed using independent t-tests. ACC n-back performance differences between stimulation groups before TMS were analyzed using linear mixed models. Here, the n-back block was included as a covariate as well as WM-load, sex, and age.

## Results

### N-back performance before TMS

First, we analyzed the n-back performance before TMS. There was a significant main effect of load (0-, 2-, 3-back) (*F*(2,672) = 343.35, *p* < 0.0001) as well as block (*F*(1,672) = 22.50, *p* < 0.0001) on ACC (ACC: hits plus correct rejections divided by the total number of letters shown), but no stimulation group effect (*F*(1,36) = 0.01, *p* = 0.92). Regarding load, *post-hoc* tests revealed a better performance for ACC 0-back (0-back mean over all blocks 0.97 ± 0.04 [SD]) than for ACC 2-back (2-back mean over all blocks 0.86 ± 0.09 [SD]; *F*(1,434) = 320.76, *p* < 0.0001), and better performances for ACC 2-back than for ACC 3-back (3-back mean over all blocks 0.80 ± 0.09 [SD]; *F*(1,436) = 75.76, *p* < 0.0001). The main effect of block on ACC indicated a significant performance increase over all 6 blocks, which indicates a learning effect. Furthermore, no significant differences in ACC before TMS were found between stimulation groups (sham cTBS, active cTBS) when each load was analyzed separately (all *p* > 0.34), and also no significant differences in the last n-back block before TMS (all *p* > 0.12). Moreover, the sham and active cTBS group did not differ on the VAS for anxiety, or STAI at any timepoint, rMT, age, handedness, time of day, or motivation (all *p* > 0.26). Furthermore, there was no association between the de facto stimulation group and the subjects' believe about group allocation (*χ* ^*2*^ (2) = 2.28, *p* = 0.43).

### N-back performance after TMS

To illustrate the influence of TMS on WM performance change, we calculated for each block the delta values. The delta values correspond to the difference in task performance between the last block before TMS and the performances after TMS (see Methods). There was no significant three-way interaction between WM-load (0-, 2-, 3-back), stimulation group (sham cTBS, active cTBS) and n-back block on delta ACC n-back performance (*F*(2,659) = 1.62, *p* = 0.20). Notably, we found a significant interaction effect between WM-load and stimulation (sham cTBS, active cTBS) on delta ACC (*F*(2,664) = 9.04, *p* = 0.0001). To determine the source of significant interaction, we applied *post-hoc* tests for each WM-load separately that revealed an impairing effect of active cTBS on delta 2-back, which survived Bonferroni correction (*F*(1,36) = 9.71, *p* = 0.0036, see Table 1). No significant main effect of stimulation on delta ACC 3-back (*F*(1,36) = 0.06, *p* = 0.81) or delta ACC 0-back performance (*F*(1,35) = 2.44, *p* = 0.13) was detected. To take into account the subjects’ response bias, we further investigated the delta d-prime 2-back performance. Again, there was a significant effect of stimulation on delta d-prime 2-back (*F*(1,36) = 8.68, *p* = 0.0056)) but not on delta d-prime 3-back (*F*(1,36) = 1.12, *p* = 0.30)) or delta d-prime 0-back (*F*(1,35) = 2.7, *p* = 0.11)). When we used the raw ACC values, instead of the delta ACC, and included the last 2-back performance before TMS as an additional covariate, there was again a significant main effect of stimulation in both measures of 2-back performance (ACC: *F*(1,35) = 5.64, *p* = 0.02; d-prime: *F*(1,35) = 5.77, *p* = 0.02). The stimulation effect on 2-back performance was independent of sex (no significant interaction between sex and stimulation on delta ACC 2-back *F*(1,35) = 0.13, *p* = 0.72) or age (no significant interaction between age and stimulation on delta ACC 2-back *F*(1,35) = 3.39, *p* = 0.07).

**Table 1:** Treatment effects on n-back performances independent of WM-load load and *Post-hoc* tests for each WM-load separately.

Variable of interest	WM-load* stimulation	ME N-back load	ME WM-load	ME stimulation	Age*load / ME age	Sex*load / ME sex
Delta ACC	*p* = 0.0001*	*p* = 0.005	*p* = 0.03	*p* = 0.80	*p* <0.0001	*p* < 0.0001
Delta ACC 0-back			*p* = 0.13	*p* = 0.14	*p* = 0.89	*p* = 0.0039
Delta ACC 2-back			*p* = 0.0036*	*p* = 0.07	*p* = 0.0039*	*p* = 0.0039*
Delta ACC 3-back			*p* = 0.81	*p* = 0.60	*p* = 0.77	*p* = 0.26

*Note*. * *p* <. 017 representing Bonferroni corrected α level for the *post-hoc* tests. ME = Main effect. There was a significant interaction between load and stimulation on delta ACC n-back performance. The interaction of age and load as well as sex and load were included as covariates. *Post-hoc* tests revealed a significant stimulation effect on 2-back performance only. For the *Post-hoc* tests only the main effects for sex and age were included as covariates, but not the interaction terms WM-load by sex and WM-load by age.

Furthermore, we tested if the stimulation effect on delta ACC 2-back performance was due to confounding variables, like VAS for anxiety or STAI at any time point, time of day, rMT, handedness, or motivation. The stimulation effect on delta ACC 2-back performance was still detectable when entering the covariates separately into the model or when entering all covariates together (all *p*_*stimulation*_ < 0.007). Moreover, calculating the average of the delta values of all 6 blocks after TMS revealed again a stimulation effect on mean delta ACC 2-back performance (*F*(1,36) = 9.72, *p* = 0.0036).

**Fig 2:**
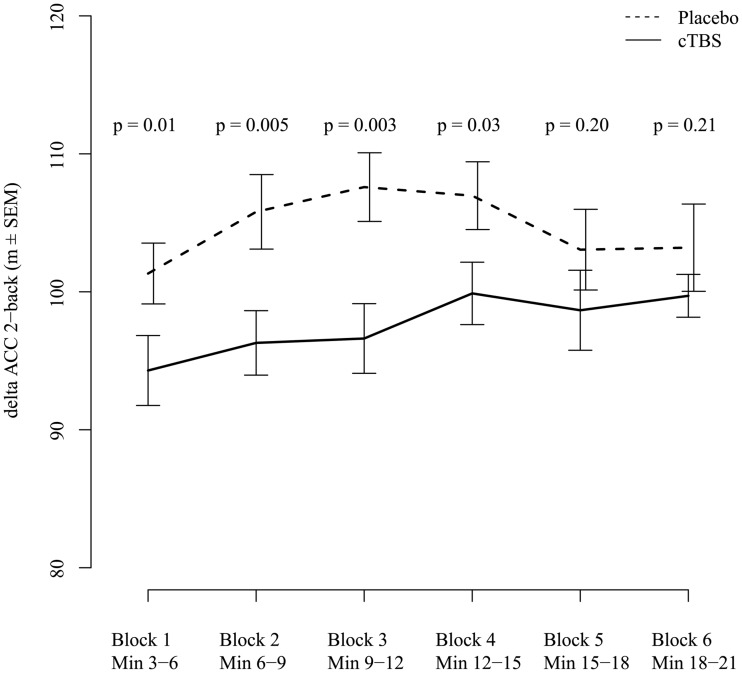
Stimulation effects on delta ACC 2-back, separately for each block after TMS. A nominal significant effect of stimulation could be detected up to block four, corresponding to 15 min after TMS (including 3 min for participant reallocation to the computer screen after TMS). Values higher than 100 indicate an improved performance compared to the last block before TMS, while values below 100 indicate decreased performance.

To investigate the time-dependency of the TMS influence on delta ACC 2-back performance, we analyzed each block separately. A nominally significant impairing effect of active stimulation on delta ACC 2-back performance could be detected in the first 4 blocks (block1: *F*(1,36) = 6.82, *p* = 0.01; block2: *F*(1,36) = 9.11, *p* = 0.005; block3: *F*(1,36) = 10.36, *p* = 0.003; block4: *F*(1,35) = 5.21, *p* = 0.03), but not any more in block5 (*F*(1,36) = 1.68, *p* = 0.20) and block6 (*F*(1,36) = 1.59, *p* = 0.21). One n-back block lasted 3 min; therefore the differences in performance between stimulation groups lasted for about 15 min (see Fig. 2).

## Discussion

We investigated the influence of cTBS applied over the left DLPFC on a WM task (n-back letter task) with 3 different cognitive loads (0-, 2-, 3-back). Remarkably, TMS only decreased medium load WM performance significantly, i.e. 2-back WM performance. This effect was present even after controlling for state anxiety, time of day, rMT, handedness, and motivation and lasted for approximately 15 min after stimulation.

Interestingly, 3-back performance was not affected by TMS. A reason could be that the TMS target (i.e. the coordinates for stimulation) was derived from an fMRI study using the 2-back task. It is therefore possible that the used TMS target was not optimal for the 3-back task. In addition to the coordinates for stimulation, other factors may have contributed to different TMS effects in the 2-back and 3-back task. A number of neuroimaging studies showed a relationship between brain activation and WM-load. These studies are indicating an enhanced activation in WM-related regions with increased load during task performance (for a review see: [11, 24]) as well as involvement of the WM-related network during task preparation as a function of expected WM-load [25]. Moreover, increased WM-load was found to modulate connectivity between regions in the WM network during task performance [26, 27]. A study investigating 3 different n-back levels with positron emission tomography described bigger activation clusters in the 3-back versus control contrast as compared to the size of the activation cluster in the 2-back versus control contrast, while the dimensions of the clusters in a 1-back versus control contrast were the smallest [28]. Thus, due to a bigger activation area in the 3-back than in the 2-back task, TMS might have failed to influence 3-back performance.

On the whole, the present result concerning the duration of the aftereffect might yield valuable information, especially for the planning of future offline TMS-fMRI studies. Due to subject positioning and calibration measures at the beginning of fMRI experiments possibly lasting 10 min depending on the exact procedure, the duration of the TMS aftereffect found in this study may not be sufficient to guarantee a TMS effect lasting throughout an offline TMS-fMRI study.

Since combined TMS-fMRI studies are of great scientific interest and might provide important insights, the search for TMS protocols inducing longer aftereffects is of importance. However, simply enhancing the number of pulses and therefore the duration of a cTBS train was found to invert the inhibitory effect into a facilitating one [29]. In contrast, applying a second cTBS train after a delay of 10 to 15 min was not only found to prolong the inhibitory aftereffect duration [30, 31], but also seems to be more resistant against de-depression [32], i.e. more resistant to reversal. Therefore, applying multiple cTBS trains, which are separated in time, might provide a way to robustly induce longer lasting aftereffects than one cTBS train alone.

Mottaghy et al. [12] demonstrated an impairing effect of 1 Hz rTMS on a delayed stimulus recognition task that lasted for about 5 min. A reason for the longer lasting effect in our study could be the use of cTBS stimulation, as there is some evidence for prolonged aftereffect durations of TBS paradigms as compared to 1 Hz rTMS. In direct comparison with 1 Hz rTMS, cTBS affected eye saccade latencies for up to 30 min, while the effect of 1 Hz rTMS lasted about 8 min [10]. However, it is possible that the cTBS aftereffect duration may vary across phenotypes.

In conclusion, the present findings suggest that cTBS applied over the left DLPFC affects WM depending on the load and has limited aftereffect duration. These results may add to the understanding of human cognitive processes and have potentially important clinical implications, as WM deficits are a key component of neuropsychiatric disorders, such as schizophrenia [33], bipolar disorders [34], and attention deficit hyperactivity disorder [35]. Moreover, it has important implications for the design of future TMS studies.
