# Correction: Continuous Theta Burst Stimulation over the Left Dorsolateral Prefrontal Cortex Decreases Medium Load Working Memory Performance in Healthy Humans

**DOI:** 10.1371/journal.pone.0146737

**Published:** 2016-01-05

**Authors:** Nathalie Schicktanz, Matthias Fastenrath, Annette Milnik, Klara Spalek, Bianca Auschra, Thomas Nyffeler, Andreas Papassotiropoulos, Dominique J.-F. de Quervain, Kyrill Schwegler

In Table 1, the headings for ME WM-load, ME stimulation and ME block do not accurately appear. Please see the corrected Table 1 here.

**Table 1:** Treatment effects on n-back performances independent of WM-load load and *Post-hoc* tests for each WM-load separately.

Variable of interest	WM-load * stimulation	ME WM- load	ME stimulation	ME block	Age*load / ME age	Sex*load / ME sex
Delta ACC	*p* = 0.0001*	*p* = 0.005	*p* = 0.03	*p* = 0.80	*p* <0.0001	*p* < 0.0001
Delta ACC 0-back			*p* = 0.13	*p* = 0.14	*p* = 0.89	*p* = 0.0039
Delta ACC 2-back			*p* = 0.0036*	*p* = 0.07	*p* = 0.0039*	*p* = 0.0039*
Delta ACC 3-back			*p* = 0.81	*p* = 0.60	*p* = 0.77	*p* = 0.26

*Note*. * *p* < .017 representing Bonferroni corrected α level for the *post-hoc* tests. ME = Main effect. There was a significant interaction between load and stimulation on delta ACC n-back performance. The interaction of age and load as well as sex and load were included as covariates. *Post-hoc* tests revealed a significant stimulation effect on 2-back performance only. For the *Post-hoc* tests only the main effects for sex and age were included as covariates, but not the interaction terms WM-load by sex and WM-load by age.
